# Nicotine Dependence and Alcohol Problems from Adolescence to Young Adulthood

**DOI:** 10.21767/2472-5048.100009

**Published:** 2016-04-25

**Authors:** Lisa Dierker, Arielle Selya, Jennifer Rose, Donald Hedeker, Robin Mermelstein

**Affiliations:** 1 Department of Psychology, Wesleyan University, Middletown, CT, USA; 2 University of North Dakota, School of Medicine and Health Sciences, USA; 3 Department of Public Health Sciences, University of Chicago, Chicago, USA; 4 Institute for Health Research and Policy, University of Illinois at Chicago, Chicago, USA

**Keywords:** Nicotine, Alcohol problems, Mixed-effects regression model

## Abstract

**Background:**

Despite the highly replicated relationship between symptoms associated with both alcohol and nicotine, little is known about this association across time and exposure to both drinking and smoking. In the present study, we evaluate if problems associated with alcohol use are related to emerging nicotine dependence symptoms and whether this relationship varies from adolescence to young adulthood, after accounting for both alcohol and nicotine exposure.

**Methods:**

The sample was drawn from the Social and Emotional Contexts of Adolescent Smoking Patterns Study which measured smoking, nicotine dependence, alcohol use and alcohol related problems over 6 assessment waves spanning 6 years. Analyses were based on repeated assessment of 864 participants reporting some smoking and drinking 30 days prior to individual assessment waves. Mixed-effects regression models were estimated to examine potential time, smoking and/or alcohol varying effects in the association between alcohol problems and nicotine dependence.

**Findings:**

Inter-individual differences in mean levels of alcohol problems and within subject changes in alcohol problems from adolescence to young adulthood were each significantly associated with nicotine dependence symptoms over and above levels of smoking and drinking behaviour. This association was consistent across both time and increasing levels of smoking and drinking.

**Conclusions:**

Alcohol related problems are a consistent risk factor for nicotine dependence over and above measures of drinking and smoking and this association can be demonstrated from the earliest experiences with smoking in adolescents, through the establishment of more regular smoking patterns across the transition to young adulthood. These findings add to accumulating evidence suggesting that smoking and drinking may be related through a mechanism that cannot be wholly accounted for by exposure to either substance.

## Introduction

One of the most potent risk factors consistently implicated in both the etiology of smoking behaviour and the subsequent development of nicotine dependence is the use of alcohol. Research within adolescence and young adulthood has shown that drinking is associated with the initiation and escalation of smoking and vice versa [1]. Notably, discussion of the mechanism of association between smoking and alcohol use has largely focused on the role of alcohol in elevating an individual’s probability of smoking exposure, including increasing the likelihood of initiation, promoting earlier onset, and/or influencing the number of cigarettes or persistence of smoking. In other words, alcohol use is believed to promote increases in smoking exposure that in turn cause physiological adaptations leading to nicotine dependence symptoms [2].

Though alcohol use is consistently linked to several smoking-related outcomes, an alternate line of emerging evidence suggests that in addition to the use of alcohol promoting exposure to cigarettes, and vice versa, alcohol-related symptoms, associated with both abuse and dependence, may be independently associated with nicotine dependence, over and above one’s level of smoking or drinking per se [3–6]. For example, an investigation of young adult smokers from the National Epidemiologic Study of Alcohol and Related Conditions (NESARC) demonstrated that daily smokers with alcohol dependence were at increased risk for nicotine dependence when examining rates of nicotine dependence across the continuum of daily smoking behaviours [3]. Individuals with a lifetime diagnosis of alcohol dependence showed higher rates of nicotine dependence at each level of daily smoking, ranging from 1 to 5 cigarettes per day to well over a pack per day, compared to individuals without a history of alcohol dependence. Further, the prevalence of nicotine dependence among those with alcohol dependence was high (~80%) even at low levels of smoking (e.g., 1 to 5 cigarettes per day) [3].

More recent evidence based on data from the National Household Survey of Drug Use and Health, showed that both alcohol abuse and alcohol dependence were associated with increased likelihood of tolerance for nicotine. Further, alcohol dependence, but not abuse was associated with nicotine related symptoms of withdrawal, craving and worry about running out of cigarettes [6]. Finally, based on a 4-year longitudinal follow-up of adolescents at risk for chronic smoking behaviour, we have previously demonstrated that among novice smokers at entry into the study, the association between alcohol-related problems at baseline and smoking frequency at the 4 year follow-up could be largely explained by experiences of nicotine dependence symptoms rather than directly through measures of smoking or drinking behaviour [5].

Discussion of the mechanism that may help to explain the association between alcohol related symptoms and nicotine dependence has, to date, largely focused on the role of alcohol use in elevating one’s probability of smoking (i.e. increasing the likelihood of initiation, promoting earlier onset, and/or influencing the number of cigarettes or persistence of smoking) [2]. The aforementioned evidence, independently linking alcohol symptoms and related problems to nicotine dependence, supports an alternate hypothesis that recognizes symptoms related to alcohol use as a sign or signal for nicotine dependence across a potentially wide range of smoking behaviour. Yet, how wide might this range be? For example, is it inclusive of even the very first experiences with cigarettes? Further, do alcohol related problems as a signal of nicotine dependence sensitivity necessarily functions consistently across time and developmental stage? Available research has largely documented static, between-subjects relationships rather than exploring the developmental growth and change in the association between symptoms related to alcohol use and smoking within individuals and across the period of greatest risk for the escalation of both smoking and drinking behaviours.

The present study sought to begin to fill this gap by examining the dynamic, longitudinal relationships between alcohol problems and nicotine dependence. Specifically, we investigate whether there are variations in the association between alcohol-related problems and emerging nicotine dependence based on time, and/or exposure to cigarettes and alcohol. We ask: (1) Are alcohol related problems independently associated with emerging nicotine dependence after accounting for both smoking and drinking behaviour? (2) Does this relationship vary across levels of smoking or drinking? and; (3) Does the relationship vary across the transition from adolescence to young adulthood? To address these questions, we rely on data from an on-going longitudinal sample recruited during adolescence and followed through the transition to young adulthood.

## Methods

### Participants

The sample was drawn from the Social and Emotional Contexts of Adolescent Smoking Patterns (SECASP) Study, which has been described elsewhere [7]. All 9th and 10th grade students at 16 Chicago-area high schools completed a brief screener survey of smoking behaviour (N = 12,970). All students who reported 1) smoking in the past 90 days and smoking <100 cigarettes/ lifetime, 2) smoking in the past 90 days and smoking >100 cigarettes/lifetime, or 3) smoking <100 cigarettes/lifetime, but not smoking in the past 90 days, were invited to participate, as were random samples of never-smokers. Only those who had already reached daily smoking levels were excluded from the study, given our interest in explaining the trajectories toward chronic, daily use over time. Of the 3654 students invited, 1263 agreed to participate and completed the baseline measurement 2 months after screening.

Following the baseline assessment, 5 additional assessment waves that included identical measures of smoking, drinking, alcohol problems and nicotine dependence occurred at 6-, 15-, 24-, 60- and 72 months. All procedures received approval from the University of Illinois at Chicago IRB. Written informed consent was obtained from the parents or guardians of the adolescents and each adolescent provided their assent to participate in the study. For assessment following each participant’s 18th birthday, informed consent was directly obtained. Retention at 72 months was 1068 participants (retention 84.6%, dropout 15.4%). The present analyses focused on participant level smoking and drinking observations across the multiple assessment waves that included reports of some smoking and drinking in the months prior to each assessment (n = 864 participants, contributing 2470 smoking and drinking observations). The mean age of this sample when recruited for the study was 15.7 years (s.d. 0.62). Fifty-five percent (n=479) were male, 58.9% (n=512) White, 14.4% (n=125), Black and 18.6% (n=162) were Hispanic.

### Measures

#### Smoking

Smoking behaviour for the present analyses was measured at the baseline, 6-, 15-, 24-, 60- and 72-month assessment waves with two items. “About how many cigarettes have you smoked in your entire life (500 or more, 100 or more cigarettes, 26 to 99 cigarettes, 16 to 25 cigarettes, 6 to 15 cigarettes, 2 to 5 cigarettes, 1 cigarette, or 1 or more puffs, but never a whole cigarette)?” and “Have you ever smoked cigarettes on a daily basis (i.e. At least 30 days when you smoked every day or nearly every day)?”

#### Nicotine dependence

Nicotine dependence was assessed at the baseline, 6-, 15, 24-, 60- and 72-month follow-up assessments with a shortened version of the nicotine dependence syndrome scale (NDSS) [8] modified for use with adolescents. The full NDSS scale was reduced to 10 items based on psychometric analyses conducted on an adolescent sample [9], retaining those items reflecting mainly drive and tolerance from the original NDSS. Research supports the reliability, stability, construct validity, and predictive validity of the NDSS for use with adolescents [10,11] and the modified version demonstrated strong internal consistency with the current sample (coefficient alpha = .93). Items in the current study were answered on a four-point Likert-type scale, ranging from 0 (not at all true) to 3 (very true), and were summed into a total NDSS score.

#### Drinking

Current alcohol use frequency and quantity was measured at the baseline, 6-, 15-, 24-, 60- and 72-month assessment waves. Because of changes in reference duration across different assessment waves from 3 months to 12 months for the alcohol frequency item “how often did you drink”, number of drinks consumed on days drinking was used as the primary measure of drinking amount in the present analyses. The questions, “when you drink alcohol, how much do you usually have at one time, on average” and “how many alcoholic drinks did you have on a typical day when you drank alcohol?” were recorded as number of drinks usually consumed.

#### Alcohol related problems

Problems related to alcohol use were measured at the baseline, 6-, 15, 24-, 60- and 72-month follow-up assessments. The ARPS scale [12–14] consisted of dichotomous endorsement of 6 items in the past year as a result of drinking alcohol. Items included 1) had a problem with, or complaints from, your family and friends; 2) been in trouble at school or work (for example, missing school or losing a job); 3) been in trouble with the police; 4) had an accident or injury; 5) awakened the morning after and found you couldn’t remember things that had happened the night before; and 6) ended up drinking more than you had expected to when you began.

#### Other tobacco use

Other tobacco use was measured at the baseline, 6-, 15, 24-, 60- and 72-month follow-up assessments with the questions, During the past 30 days, on how many days did you (a) use chewing tobacco, snuff or dip; (b) smoke cigars, cigarillos or little cigars; (c) smoked bidis (small, thin, hand-rolled cigarettes wrapped in tendu or temburni leaf) or (d) smoked kreteks (cigarettes typically containing a mixture of tobacco and cloves)? At the 60 and 72 month follow-up assessments, other tobacco use was measured by the questions, During the past 30 days, on how many days did you (a) use chewing tobacco, snuff or dip; (b) smoke cigars; (c) use snus (a moist powder, smokeless tobacco); (d) use e-cigarettes or (e) smoke a hookah? Responses at each assessment wave were dichotomized into any other tobacco use vs. no other tobacco use.

#### Other drug use

Other drug use was measured at the baseline, 6-, 15, 24-, 60- and 72-month follow-up assessments with the individual questions, during the past 3 months, how often did you smoke marijuana, use cocaine, amphetamines, hallucinogens, inhalants, or steroids, take drugs with a needle or use prescription drugs not meant for you. Due to the low base rates of all but marijuana use, responses were dichotomized into two variables, any marijuana use vs. no marijuana use and other drug use vs. no other drug use.

### Analyses

Using nicotine dependence symptom scores from the NDSS as the outcome, between-subjects (static) effects and time-varying effects of alcohol use problems, usual number of drinks consumed, and number of lifetime cigarettes smoked at each assessment were examined, while controlling for time varying effects of daily smoking, other tobacco use, marijuana use and other drug use, and subject-level effects of age, ethnicity (White vs. non-White) and gender measured at baseline. Mixed-effects regression models (i.e. HLM or multilevel models), which include both fixed effects of variables and random effects to account for the repeated measurements of participants over time, were run using SAS PROC MIXED. Random effects included the intercept (allowing individual differences in baseline nicotine dependence), time trends (allowing individual differences in the rate of change in nicotine dependence across follow-up waves), smoking level (allowing individual differences in the rate of change in nicotine dependence with varying exposure to smoking) and usual number of drinks consumed (allowing individual differences in the rate of change in nicotine dependence with varying exposure to alcohol) with an unstructured covariance structure.

Nicotine dependence symptoms (outcome), alcohol problems, usual number of drinks consumed, number of lifetime cigarettes smoked, daily smoking, other tobacco, marijuana and other drug use were all time-varying, while age, ethnicity and gender were static and derived from the baseline reports. To disentangle the contribution of within-subjects changes in smoking, drinking and alcohol problems and between-subjects variability in predicting nicotine dependence, mixed effects regression models were estimated to simultaneously account for both an individual’s mean level of smoking, drinking and alcohol problems across time (i.e. between-subjects effects) as well as the difference at each assessment wave between an individual’s mean level of smoking, drinking and alcohol problems and their current level at each assessment (i.e. within-subjects effects) [15]. Both time-varying and smoking and drinking exposure-varying effects were investigated by including individual 2-way interaction terms between alcohol problems with time, smoking, and drinking.

## Results

Average lifetime smoking, usual number of drinks consumed, alcohol problems, and nicotine dependence symptoms at each assessment wave (over time), are shown in Table 1. Positive linear trends characterize average lifetime smoking levels (b=4.05, p=.0001) and nicotine dependence symptom scores (b=.05, p=.0001) between baseline and 72 months. In contrast, mean usual number of drinks showed a negative quadratic trend (b= −.0005, p=.0001) in which average number of drinks increased from baseline to 24 months, and then decreased from 24 month to 72 months. No significant linear trend was seen in average number of alcohol problems between the baseline and 72 month assessments.

The results of the mixed-effects regression model are shown in Table 2. Alcohol problems were consistently associated with the nicotine dependence symptom score, after controlling for baseline measures of gender, age, and ethnicity and time varying measures of daily smoking, other tobacco use, marijuana use and other drug use. That is, the between subjects measure of alcohol problems (mean alcohol problems across assessment waves) as well as the within-subjects measure (the difference between mean alcohol problems and current alcohol problems at each wave) were significantly and positively associated with nicotine dependence symptom scores, indicating that individuals with consistently higher alcohol problems scored consistently higher on the nicotine dependence measure (i.e. NDSS), and that within individuals, increases in alcohol problems at a particular wave are associated with increases in nicotine dependence symptoms at that wave. Notably, the associations between alcohol problems and nicotine dependence were significant even after accounting for the between-subject and within-subject measures of usual number of drinks and lifetime cigarette exposure. The within subject measure of usual number of drinks, the within and between-subject measures of lifetime cigarette exposure, as well as daily smoking, other tobacco use, marijuana use and other drug use were also found to be independently associated with nicotine dependence symptom scores.

The relationship between alcohol problems and nicotine dependence did not vary by time, or by smoking or drinking measures as was observed by the non-significant interactions tested in the mixed-effects model.

## Discussion

Despite considerable evidence supporting an alcohol-smoking link, the mechanisms underlying this association remain relatively unclear. The present study sought to investigate this relationship from the perspective of emerging nicotine dependence symptoms and within a developmental context including the transition from adolescence to young adulthood. Three major findings emerged. First, after statistical control for time-varying measures of nicotine exposure (i.e. lifetime cigarette smoking, current daily smoking and other tobacco use) and usual number of drinks consumed, alcohol problems were positively and significantly associated with nicotine dependence symptoms. Second, this association was significant when considering both between-subject differences as well as within-subject changes in alcohol problems and nicotine dependence symptoms across time. Third, the association between alcohol problems and nicotine dependence symptoms was consistent across time (i.e. from adolescence to young adulthood) and did not differ based on measures of smoking exposure or drinking behaviour.

The link between alcohol use and smoking has most often been discussed in terms of the role of each behaviour in increasing risk of exposure to the other substance [2] or as general markers of risk taking [16]. In contrast, the present findings suggest that alcohol-related problems, independent of substance use exposure that includes not only smoking and drinking, but also other tobacco use, marijuana use and other drug use is associated with nicotine dependence symptoms. It is possible that neurobiological changes related to the emergence of alcohol-related problems may increase the propensity for similar changes related to nicotine dependence. For example, preclinical data suggest animals that develop tolerance to the effects of one substance may also become tolerant to the effects of the other as demonstrated with behavioural data [17]. Alternately, a third variable may be related to risk for both alcohol problems and nicotine dependence [18]. For example, selectively breeding mice for sensitivity to one substance results in animals with heightened sensitivity to the other substance, suggesting that shared genetic risk factors [19–24]. Overall, the present findings confirm that alcohol-related problems likely play a role in the experience of nicotine dependence symptoms either as a cause and/or signal of sensitivity to nicotine dependence symptoms, over and above exposure to smoking, drinking or other types of substance use.

To our knowledge, this is the first study to demonstrate an association between changes in alcohol problems and nicotine dependence symptoms (i.e. within subject effects) across time, an association that was significant from adolescence to young adulthood. Further, the significant associations between measures of smoking, drinking, other substance use behaviour and nicotine dependence symptoms, independent of alcohol use problems, suggests that an individual’s cumulative exposure to substances across time, rather than time per se, also plays a significant role in development of nicotine dependence symptoms.

The current findings should be interpreted within the context of study limitations. Because we were interested in the association between alcohol problems and nicotine dependence, data for these analyses included individual observations for youth who had smoked and drank in the months prior to an assessment wave. Thus, while the findings demonstrate that increases in nicotine dependence are associated with increases in alcohol problems and vice versa, we were unable to evaluate this question among those who had quit smoking or drinking. We also do not provide evidence regarding the temporal association between alcohol problems and nicotine dependence (i.e. which comes first) given our focus on the time varying nature of the cross-sectional relationship between these two constructs. Additionally, these results are directly tied to the measurement of nicotine dependence according to the NDSS, and as such mainly reflect drive and tolerance dimensions. Finally, while we were able to examine an array of alcohol related problems, a more formal assessment of alcohol abuse and dependence symptoms was not available.

Despite these limitations, the current study has a number of strengths. First, it is based on one of only a few longitudinal samples available to date that includes a large group of youth at the earliest stages of smoking exposure. Further, these findings are among the first to characterize the relationship between alcohol problems and nicotine dependence across both time and levels of smoking and drinking. As such, the present study adds to accumulating evidence showing individual variability in nicotine dependence symptoms based on problems associated with alcohol use, an association that was not better accounted for by smoking or drinking behaviour. If causally associated, these findings would suggest that treatment of alcohol problems may prevent or reduce the early emergence of nicotine dependence symptoms. If instead, however, alcohol problems are a signal of sensitivity for nicotine dependence, best accounted for by a third variable, then adolescents with measurable symptoms associated with either smoking or drinking, regardless of their level of use, represent an important subgroup that may benefit from intervention [25].

## Figures and Tables

**Table 1:** Average lifetime smoking, current number of drinks consumed, alcohol problems and nicotine dependence score at each assessment wave, 1Mean of median value of the endorsed response category.

	No. Lifetime Cigarettes	Usual No. Drinks	Alcohol Problems	Nicotine Dependence
	Mean (s.d.)1	Mean (s.d.)	Mean (s.d.)	Mean (s.d.)
**Baseline**	112.4 (171.71)	5.0 (2.04)	2.0 (1.38)	7.0 (7.16)
**6 month**	168.7 (204.36)	5.4 (1.92)	2.0 (1.46)	8.0 (7.68)
**15 months**	206.9 (219.01)	5.5 (1.94)	2.0 (1.43)	9.0 (8.29)
**24 months**	242.3 (220.23)	5.8 (1.78)	2.1 (1.59)	9.5 (7.90)
**60 months**	366.8 (200.73)	5.0 (1.57)	2.1 (1.44)	10.4 (8.70)
**72 months**	388.8 (187.95)	4.9 (1.62)	2.0 (1.38)	10.7 (8.60)

**Table 2:** Unstandardized fixed effects on nicotine dependence symptoms, based on results of a mixed-effects regression model.

Effect	Estimate	Standard Error	t Value	P value
**Intercept**	5.1027	3.9257	1.3	0.194
**Alcohol Problems (between subjects)**	0.4925	0.1805	2.73	0.0075
**Alcohol Problems (within subjects)**	0.3863	0.09895	3.9	0.0001
**Usual no. drinks consumed (between subjects)**	−0.0452	0.09127	−0.5	0.6214
**Usual no. drinks consumed (within subjects)**	0.212	0.0781	2.71	0.0073
**Smoking exposure (between subjects)**	0.02074	0.00119	17.49	<0.0001
**Smoking exposure (within subjects)**	0.00969	0.00095	10.25	<0.0001
**Daily smoking**	4.1632	0.3067	13.58	<0.0001
**Other tobacco use**	1.2451	0.2221	5.6	<0.0001
**Marijuana use**	0.5313	0.265	2.0	0.0476
**Other drug use**	0.5494	0.2562	2.14	0.0344
**White vs. other**	−0.2219	0.3282	−0.68	0.5005
**Gender (male vs. female)**	0.6189	0.3391	1.82	0.071
**Age**	−0.3995	0.2505	−1.6	0.1138
**time (baseline vs. 72 mo.)**	1.1155	0.4829	2.31	0.0229
**time (6 mo. vs. 72 mo.)**	0.7526	0.4591	1.64	0.1042
**time (15 mo. vs. 72 mo.)**	1.2031	0.4315	2.79	0.0063
**time (24 mo. vs. 72 mo.)**	0.7165	0.4048	1.77	0.0798
**time (60 mo. vs. 72 mo.)**	0.1138	0.3026	0.38	0.7076

## References

[1] Dierker LC, Canino G, Merikangas KR (2006). Association between parental and individual psychiatric/substance use disorders and smoking stages among Puerto Rican adolescents. Drug and Alcohol Dependence.

[2] Pomerleau O (1995). Individual differences in sensitivity to nicotine: Implications of genetic research on nicotine depenence. Behavior Genetics.

[3] Dierker L, Donny E (2008). The role of psychiatric disorders in the relationship between cigarette smoking and DSM-IV nicotine dependence among young adults. Nicotine & Tobacco Research.

[4] Dierker L, Perrine N, Clayton R (2005). Sensitivity and specificity of quantity/frequency data for identifying nicotine dependence. Nicotine & Tobacco Research.

[5] Dierker L, Selya A, Piasecki T, Rose J, Mermelstein R (2013). Alcohol problems as a signal for sensitivity to nicotine dependence and future smoking. Drug and Alcohol Dependence.

[6] Dierker LC, Rose JS, Donny E, Tiffany S (2011). Alcohol use as a signal for sensitivity to nicotine dependence among recent onset smokers. Addictive Behaviors.

[7] Dierker L, Mermelstein R (2010). Early emerging nicotine-dependence symptoms: A signal of propensity for chronic smoking behavior in adolescents. Journal of Pediatrics.

[8] Shiffman S, Waters AJ, Hickcox M (2004). The Nicotine Dependence Syndrome Scale: A multidimensional measure of nicotine dependence. Nicotine & Tobacco Research.

[9] Sterling K, Mermelstein R, Turner L, Diviak K, Flay B (2009). Examining the psychometric properties and predictive validity of a youth-specific version of the Nicotine Dependence Syndrome Scale (NDSS) among teens with varying levels of smoking. Addictive Behaviors.

[10] Clark D, Wood DS, Martin CS, Cornelius JR, Lynch KG (2005). Multidimensional assessment of nicotine dependence in adolescents. Drug and Alcohol Dependence.

[11] Sledjeski EM, Dierker LC, Costello D, Shiffman S, Donny E (2007). Predictive validity of four nicotine dependence measures in a college sample. Drug and Alcohol Dependence.

[12] Chassin L, Rogosch F, Barrera M (1991). Substance use and symptomatology among adolescent children of alcoholics. Journal of Abnormal Psychology.

[13] Colder CR, Chassin L (1999). The psychosocial characteristics of alcohol users versus problem users: Data from a study of adolescents at risk. Developmental Psychopathology.

[14] Read JP, Kahler C, Strong DR, Colder C (2006). Development and preliminary validation of the young adult alcohol consequences questionnaire. Journal of Studies on Alcohol.

[15] Hedeker D, Gibbons RD (2006). longitudinal data analysis.

[16] Selya A, Rose JS, Dierker LC, Hedeker D, Mermelstein RJ (2012). A practical guide to calculating Cohen’s f2, a measure of local effect size, from PROC MIXED Frontiers in Quantitative Psychology and Measurement. Frontiers in Quantitative Psychology and Measurement.

[17] Cohen J (1988). Statistical power analysis for the behavioral sciences.

[18] Jessor R, Jessor L (1977). Problem behavior and psychosocial development - A longitudinal study of youth.

[19] Funk D, Marinelli PW, Le AD (2006). Biological processes underlying co-use of alcohol and nicotine: Neuronal mechanisms, crosstolerance, and genetic factors. Alcohol Res Health.

[20] Li TK, Volkow ND, Baler RD, Egli Mark (2007). The biological bases of nicotine and alcohol co-addiction. Biological Psychiatry.

[21] Bien TH, Burge R (1990). Smoking and Drinking - A literature review. International Journal of Addiction.

[22] Little HJ (2000). Behavioral mechanisms underlying the link between smoking and drinking. Alcohol Res Health.

[23] Littleton J, Little H (2002). Interactions between alcohol and nicotine dependence: A summary of potential mechanisms and implications for treatment. Alcohol Clin Exp Res.

[24] Prendergast MA, Rogers DT, Barron Susan, Bardo MT, Littleton JM (2002). Ethanol and nicotine: A pharmacologic balancing act?. Alcohol Clin Exp Res.

[25] Kalman D, Kim S, DiGirolamo G, Smelson D, Ziedonis D (2010). Addressing tobacco use disorder in smokers in early remission from alcohol dependence: The case for integrating smoking cessation services in substance use disorder treatment programs. Clin Psychol Rev.

